# iMPI: portable human-sized magnetic particle imaging scanner for real-time endovascular interventions

**DOI:** 10.1038/s41598-023-37351-2

**Published:** 2023-06-28

**Authors:** P. Vogel, M. A. Rückert, C. Greiner, J. Günther, T. Reichl, T. Kampf, T. A. Bley, V. C. Behr, S. Herz

**Affiliations:** 1 Department of Experimental Physics 5 (Biophysics), Julius-Maximilians-University Würzburg, Würzburg, Germany; 2 Department of Diagnostic and Interventional Radiology, University Hospital Würzburg, Würzburg, Germany; 3 Department of Diagnostic and Interventional Neuroradiology, University Hospital Würzburg, Würzburg, Germany

**Keywords:** Imaging, Biomedical engineering, Electrical and electronic engineering, Three-dimensional imaging

## Abstract

Minimally invasive endovascular interventions have become an important tool for the treatment of cardiovascular diseases such as ischemic heart disease, peripheral artery disease, and stroke. X-ray fluoroscopy and digital subtraction angiography are used to precisely guide these procedures, but they are associated with radiation exposure for patients and clinical staff. Magnetic Particle Imaging (MPI) is an emerging imaging technology using time-varying magnetic fields combined with magnetic nanoparticle tracers for fast and highly sensitive imaging. In recent years, basic experiments have shown that MPI has great potential for cardiovascular applications. However, commercially available MPI scanners were too large and expensive and had a small field of view (FOV) designed for rodents, which limited further translational research. The first human-sized MPI scanner designed specifically for brain imaging showed promising results but had limitations in gradient strength, acquisition time and portability. Here, we present a portable interventional MPI (iMPI) system dedicated for real-time endovascular interventions free of ionizing radiation. It uses a novel field generator approach with a very large FOV and an application-oriented open design enabling hybrid approaches with conventional X-ray-based angiography. The feasibility of a real-time iMPI-guided percutaneous transluminal angioplasty (PTA) is shown in a realistic dynamic human-sized leg model.

## Introduction

Cardiovascular diseases (CVDs) are the leading cause of global mortality and a major contributor to disability^1^. Minimally invasive endovascular interventions have become an important part of treatment of patients with CVD, such as ischemic heart disease, peripheral artery disease, or stroke^2–4^. Interventional procedures utilizing catheters and guide-wires are, e.g., performed to reopen obstructed vessels or dissolve blood clots. Minimally invasive image-guided interventions typically do not require general anesthesia or large incisions, making them much safer for patients than surgery.

The rapid evolution of endovascular interventions is driven by sophisticated imaging methods with high temporal and spatial resolution as well as the development of dedicated interventional instruments. X-ray fluoroscopy and digital subtraction angiography (DSA) are currently the standard imaging modalities for these procedures. However, X-ray-based methods are associated with radiation exposure for patients and clinical staff. In addition, iodine-containing contrast media are used, which can potentially cause acute damage of the kidneys^5^.

Magnetic Particle Imaging (MPI) is an emerging experimental imaging technique that does not involve ionizing radiation or nephrotoxic contrast media^6^. In contrast to established clinical imaging modalities such as Computed Tomography (CT), Magnetic Resonance Imaging (MRI), and X-ray, MPI is a tracer-based imaging modality. MPI uses magnetic fields to detect the spatial distribution of tracer agents composed of magnetic nanoparticles (MNPs). The concept of MPI is based on the nonlinear magnetization response of those MNPs to time-varying magnetic fields. MNP-based intravascular tracers can visualize the vasculature background-free as in DSA and have been used as MRI contrast agents in humans^7,8^. MPI features fast and sensitive imaging with a high signal-to-noise ratio (SNR)^9^ and has no depth attenuation in human tissues^10^. Due to technical reasons, MPI scanners were essentially large and stationary small animal systems with small fields-of-view (FOVs) of only a few centimeters in each dimension^11,12^. Applications in the field of CVD have been limited to initial pre-clinical phantom studies so far^13–20^.

Up-scaling of MPI scanners to human size remains challenging despite advances in hardware approaches^11,21–24^. Recently, a first human-sized MPI scanner dedicated for brain applications has been proposed, which demonstrated up-scaling with magnetic field gradients up to 0.25 T/m without sophisticated cooling^25^. To achieve higher spatial resolution, it is necessary to increase the strength of the magnetic field gradient. This comes with a higher power consumption, which increases the demands on the temperature regulation of the system and may result in a higher specific absorption rate (SAR) and peripheral nerve stimulation (PNS)^26,27^.

Another major challenge of MPI is real-time visualization, which is crucial for cardiovascular interventions. To date, many technical features required for real-time MPI-guided interventions have been demonstrated with pre-clinical scanners^13^. These include high-sensitivity detection of MNPs^9,28^, high spatial resolutions^29,30^ for fast encoding and (*in-vivo*) imaging in 2D^31,32^ and 3D^14^ as well as rapid data reconstruction and visualization with latency times below 100 ms^33^. The visualization latencies of common scanner types are often more than 2 s^34^, which would de facto preclude real-time clinical applications. Recently, however, technical improvements in scanner hardware and image reconstruction algorithms enabled in-vitro MPI-guided percutaneous transluminal angioplasty (PTA) and stenting in real-time^15,16^.

The major obstacle to the translation from pre-clinical to clinical imaging-guided interventions and to the use of MPI on a human scale has been the lack of a scanner with sufficient bore size and FOV operating with real-time visualization. The crucial next step toward potential MPI-guided endovascular interventions in a clinical setting would be the development of dedicated human-sized MPI scanners. Such a scanner should provide real-time visualization speed, high sensitivity and spatial resolution for precise instrument guidance and visualization of vascular structures, while ensuring good access to the patient and taking into account the SAR and PNS limitations.

Here, we present a portable human-sized interventional MPI scanner (iMPI) designed for real-time endovascular interventions of human extremities. The field generator design combines the highly sensitive field-free line (FFL) encoding scheme^35^ with a fast dynamic linear gradient array (dLGA)^36,37^ enabling real-time imaging of very large FOVs. In 3D-printed realistic human vessel phantoms, the iMPI scanner visualized different vascular pathologies such as aneurysms and stenoses. Real-time angiography and image-guided PTA were feasible. A dedicated ‘X-ray-window’ in the MPI scanner setup allowed simultaneous imaging with a conventional DSA / fluoroscopy system.

## Results

### iMPI scanner setup

**Figure 1:**
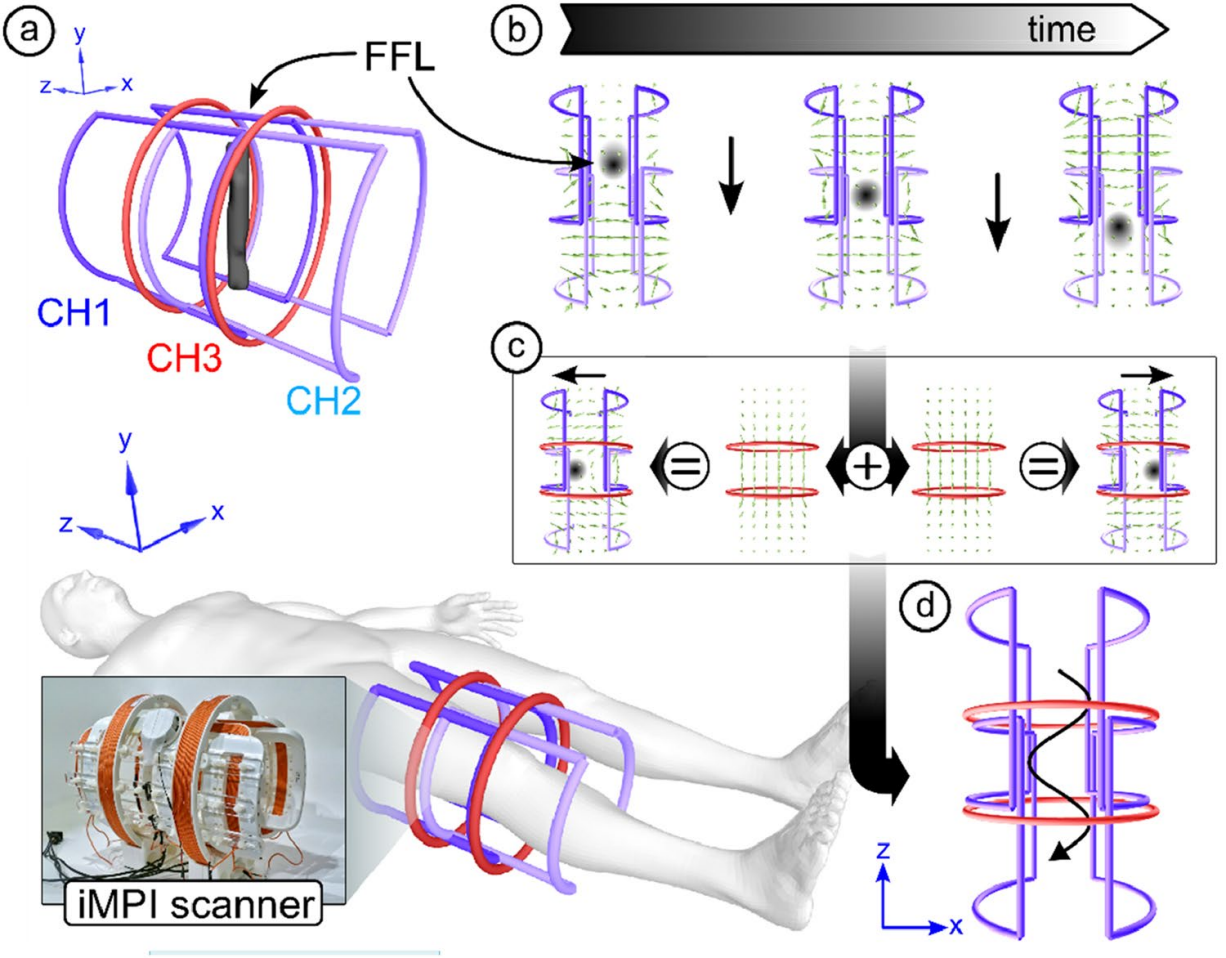
Sketch of the concept for compact human-sized MPI scanners using a field-free line (FFL). (**a**,**b**) Two pairs of saddle-coils are driven with a frequency *f*_1_ = *f*_2_ = 60 Hz and a phase shift of 90 degrees resulting in a traveling FFL along the symmetry axis of the scanner. (**c**) Two additional solenoid coils in Helmholtz configuration (CH3) are used for FFL displacement within the X–Y-plane. (**d**) Running CH3 with a frequency *f*_3_ ≫ *f*_1_, the FFL is steered along a sinusoidal trajectory through the FOV generating 2D projections.

The iMPI concept follows a minimalistic design using three independent transmit chains for the generation of fast 2D projection imaging using a field-free line (FFL) encoding scheme^35^ as sketched in Fig. 1a. Two overlapping saddle-coil pairs in Helmholtz configuration (CH1 and CH2) operating at a frequency of *f*_1_ = *f*_2_ = 60 Hz and a phase shift of 90 degrees, generate a field-free line traveling along the symmetry axis (Fig. 1b). This dynamic generation of the FFL follows the traveling wave approach, which combines both the generation of an encoding scheme (FFP or FFL) and its movement along one scanner axis^36,37^. An additional pair of solenoids (CH3) operating with a frequency of *f*_3_ = 2480 Hz steers the FFL along a sinusoidal trajectory through the desired FOV generating 2D projections (x–z plane) of the MNP distribution (Fig. 1c,d).

The size of the scanner was chosen to allow the scanning of a human thigh. With an inner bore diameter of about 20 cm and a FOV area in form of an elliptical tube (length of about 25 cm, minor diameter of about 10 cm and major diameter of about 20 cm), the assembled iMPI scanner provides a gradient strength of up to 0.36 T/m (0.25 T/m at 70% system power) and real-time visualization with a maximum of 8 frames per second. The generated magnetic fields reach a maximum amplitude of about 35 mT (70 mT pp), which is at the limit of PNS stimulation for the desired frequency^26^ (see section PNS and SAR). The power consumption of about 14 kW can yield a critical increase in temperature during continuous imaging. This can be handled by increasing the duty-cycle and/or decreasing the gradient strength (see section power consumption). The design of the iMPI scanner was chosen to be portable and lightweight with about 10 kg (approx. 8 kg of copper, 1.2 kg PLA (polylactic acid), and 0.8 kg epoxy resin).

### Dilution series

**Figure 2:**
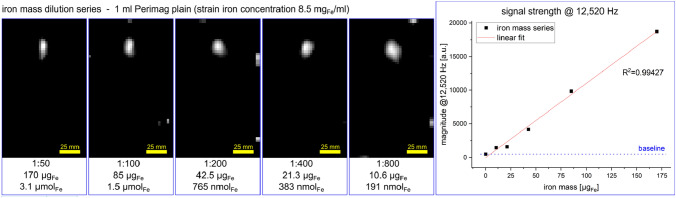
Iron mass series measurement results showing the reconstructed images of image acquisitions (50 ms) for different amount of iron mass diluted in 1 ml of water filled in a point-like sample (Eppendorf cap). The strain concentration of the used tracer (Perimag) was 8.5 mg/ml iron and was diluted in steps from 1:50 to 1:800 or 3.1 µmol Fe to 191 nmol Fe respectively. At higher dilutions, some small image artifacts appeare at the edge of the FOV, which are the result of the image-based matrix reconstruction approach. The graph on the right shows the signal strength at 12,520 Hz.

For determining the sensitivity of the iMPI scanner, a point-like sample (Eppendorf cap) filled with 1 ml of diluted Perimag (iron concentration 8.5 mg/ml, Micromod Partikeltechnologie GmbH, Germany) in steps 1:50 (3.1 µmol Fe), 1:100 (1.5 µmol Fe), 1:200 (765 nmol Fe), 1:400 (383 nmol Fe), and 1:800 (191 nmol Fe) were measured (single data acquisition time of 50 ms, scanner parameters: currents *I*_CH1/2_ = 110 A, *I*_CH3_ = 75 A). Following the reconstruction process (more details are specified in the method section), for each dilution the position of the Eppendorf sample could be clearly reconstructed repetitive for each dilution (see Fig. 2). This iron concentration was far below the requirements for dose limit of 200 mg Fe (2.5 mg Fe per kg for 80 kg person)^38^. The SNR map shows the detection limit at the 5^th^ higher harmonic band (5 × *f*_3_ + 2 × *f*_1_).

### Tracking of endovascular devices

**Figure 3:**
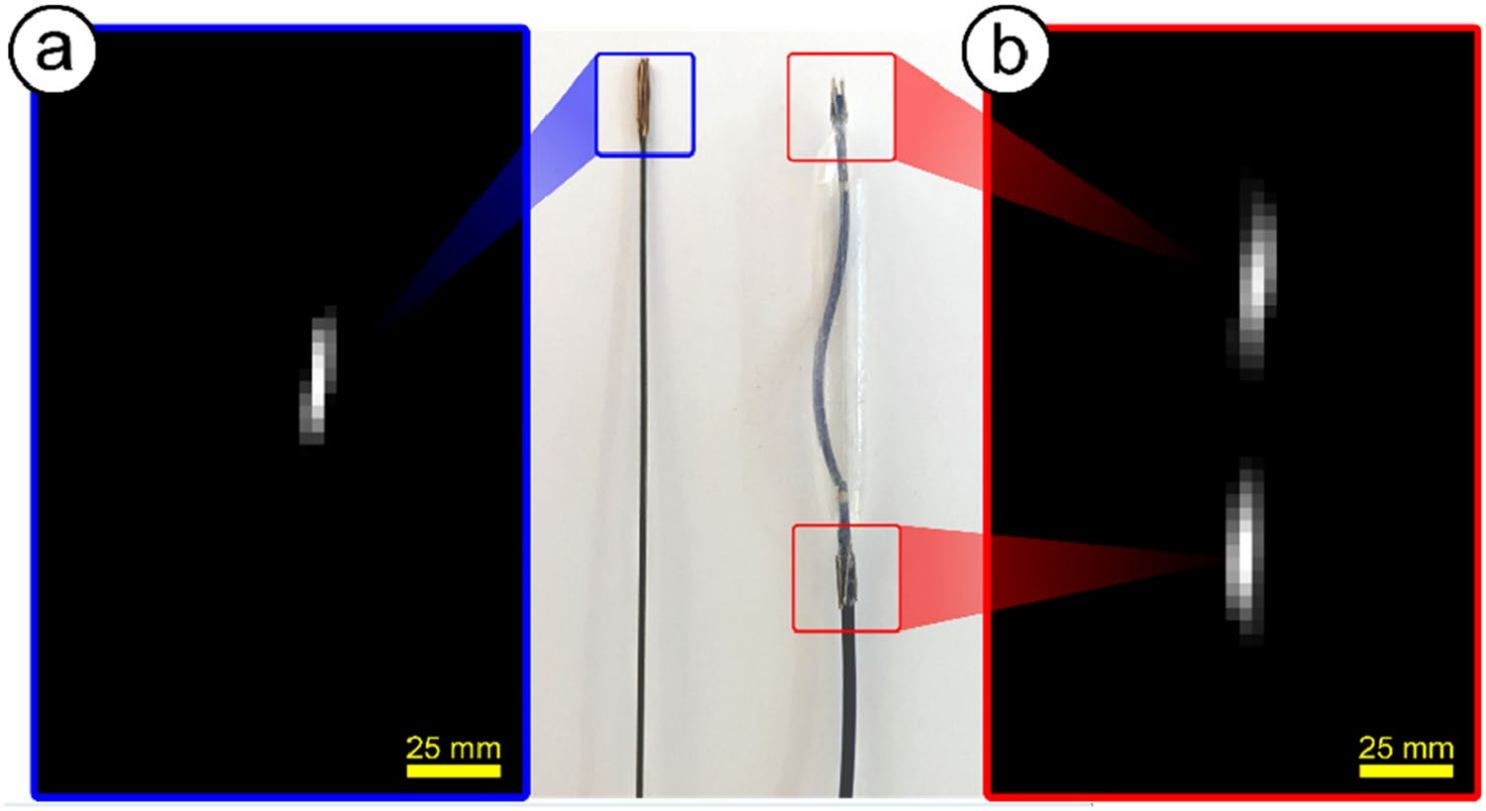
The MPI visible instruments visualized within the iMPI scanner: (**a**) shows the reconstruction of the modified conventional guide-wire with one marker and (**b**) the reconstruction of the modified balloon prepared with two markers. The photo in the middle depicts the instruments modified with an MPI visible material. The obtained SNR allowed the real-time visualization without averaging.

In Fig. 3 middle, a photo of both crucial instruments for image-guided PTA, a guide-wire and balloon catheter, modified with MPI visible material, is shown (more details are specified in the method section). For demonstration, both instruments have been measured under real-time conditions with 4 images per second (single data acquisition time of 50 ms, scanner parameters: currents *I*_CH1/2_ = 110 A, *I*_CH3_ = 75 A). The reconstructed images of the instruments after one single shot are provided on Fig. 3a,b right.

### Vessel phantoms of vascular pathologies

**Figure 4:**
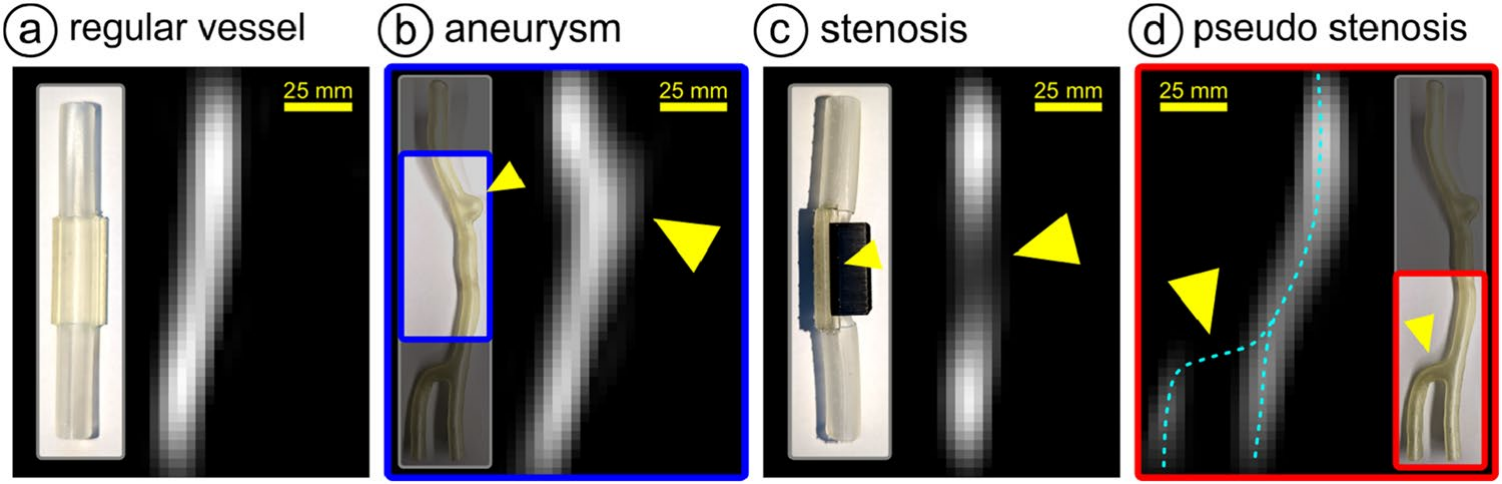
Vessel phantoms with different vascular pathologies. Photograph and iMPI visualization of (**a**) a regular vessel phantom, (**b**) an aneurysm, and (**c**) a stenosis (grade of stenosis 70%). (**d**) Shows a pseudo stenosis resulting from a reconstruction artifact. All samples were filled with Perimag (iron concentration of 8.5 mg/ml) and have been measured under real-time conditions.

In these experiments, vascular pathologies were visualized in realistic vascular models by the injection of tracer agent (more details are specified in the method section). For demonstration purposes, different vessel phantoms were visualized by real-time imaging. Figure 4 shows a normal vessel (a), an aneurysm (b), and a stenosis (c). Figure 4d shows a pseudo-stenosis because of a reconstruction artifact. The data has been acquired with single data acquisition time of 50 ms and scanner parameters using currents of *I*_CH1/2_ = 110 A, *I*_CH3_ = 75 A.

### MPI-guided percutaneous transluminal angioplasty (PTA)

**Figure 5:**
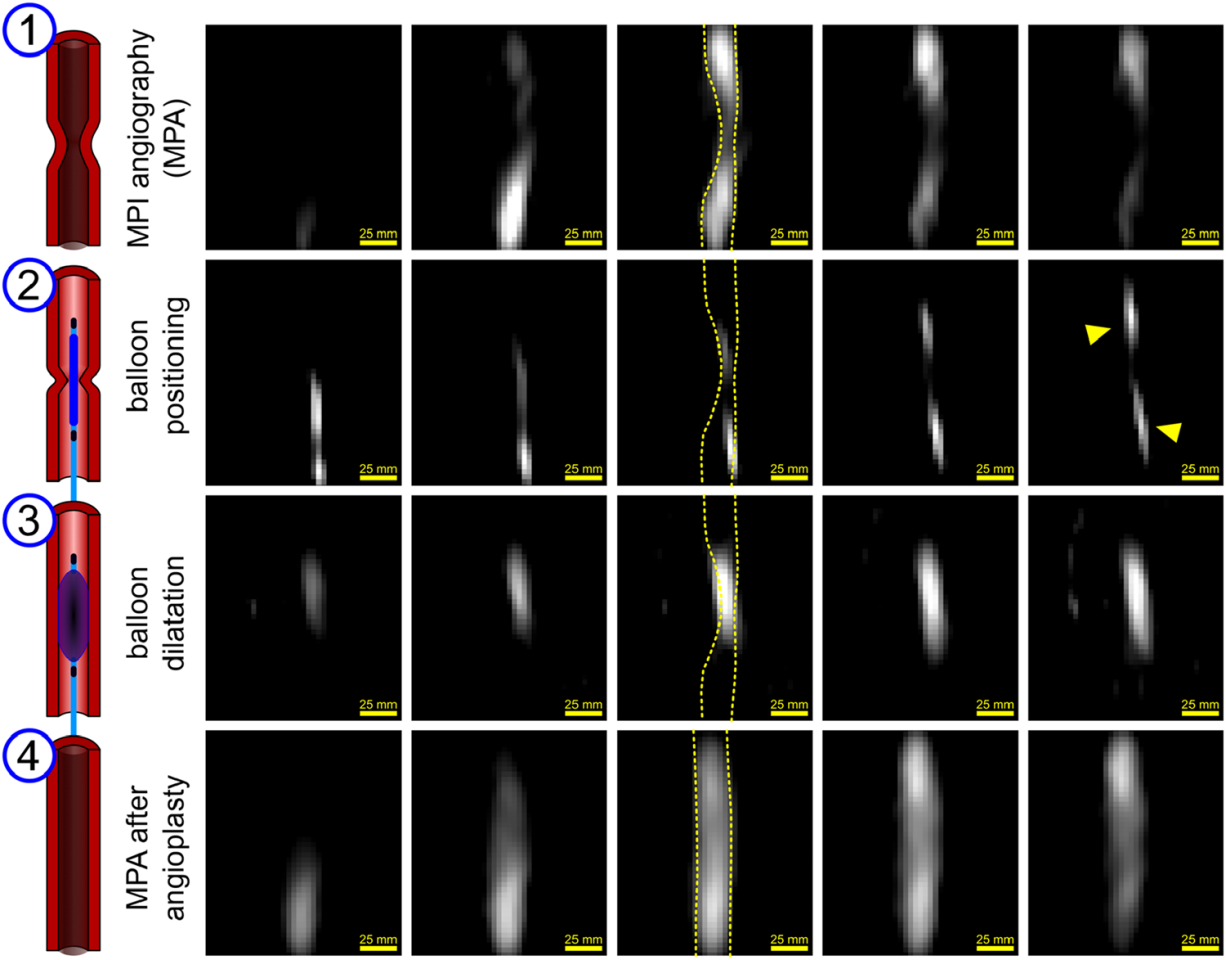
iMPI real-time visualization of an MPI guided PTA with 4 frames per second (single data acquisition time 50 ms). The covered FOV is 11 × 12 cm^2^. The peak pulsatile flow velocity in the experimental vessel system was 50 cm per second. **(1)** In a first step, the location of the experimental stenosis (grade of stenosis 70%) was determined by injecting a 1 ml Perimag (iron concentration 8.5 mg/ml) bolus (Magnetic Particle Angiography—MPA). Balloon positioning **(2)** was visualized by MPI-visible markers attached before and after the catheter-mounted balloon (bright oval signals, yellow arrows). Balloon dilation **(3)** of the stenosis was performed by inflating the balloon catheter with Perimag. Image reconstruction of **(2)** and **(3)** were performed from the same data set with different parameters (multicontrast MPI) and data subtraction. Finally, a second MPA **(4)** visualized the successful treatment of the stenosis. For animations see Supplementary files anim_PTAX.gif.

The MPI system must provide not only the capability of tracking and visualizing the required instruments but also the entire angiography steps. This requires fast and robust operation with high sensitivity. In addition, the real-time visualization with different materials (multicolor MPI^39^) can be challenging due to the use of different reconstruction parameters for different materials. In Fig. 5, several steps of the intervention procedure are shown, including the injection of a bolus of tracer agent into the stenosis phantom (angiography) before (Fig. 5 first row) and after balloon dilatation (Fig. 5 fourth row). All data have been acquired in real-time and under pulsatile flow using a gear pump to simulate the human vasculature. The data set of the balloon positioning markers and inflation process are reconstructed with different parameters for visualizing the markers and the inflation separately (Fig. 5 second & third rows). All data has been acquired under realistic conditions with 4 frames per second (single data acquisition time 50 ms, scanner parameters: currents *I*_CH1/2_ = 110 A, *I*_CH3_ = 75 A) and pulsatile flow. The black receive coil (see detailed information in the methods section) provides a FOV with a diameter of 11 cm and 12 cm in length (see Fig. 9).

### Simultaneously X-ray / MPI angiography

**Figure 6:**
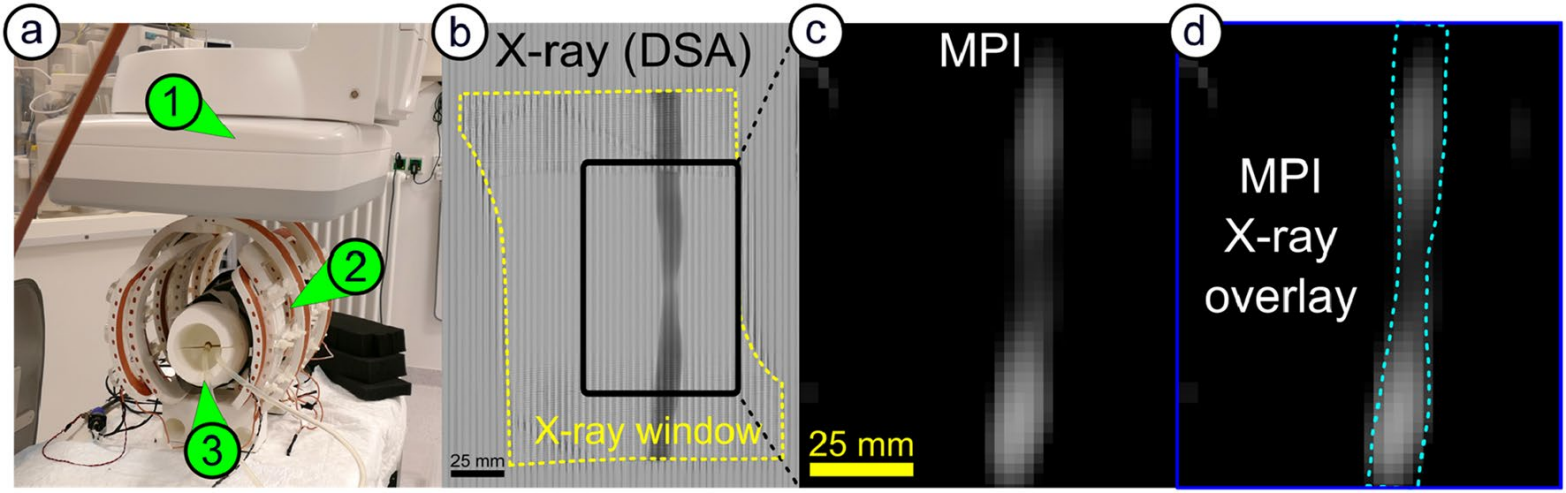
First simultaneous iMPI X-ray bolus tracking using a Perimag and iodinated contrast media mixture (ratio undiluted Perimag to iodined contrast agent of 1:1). **(a)** Image of the iMPI scanner (2) within the X-ray system (1). Simultaneous real-time visualization of the bolus through an artificial vascular stenosis in a human-size knee model (3): **(b)** X-ray and **(c)** iMPI. (d) shows an overlay of both modalities. For video see Supplementary file iMPImeetsXRay.mp4.

An application milestone on the way to a possible stand-alone iMPI-guided intervention is its use in a hybrid application together with established conventional X-ray angiography. Therefore, an "X-ray window" was integrated into the scanner setup (see receive coils in the method section). First hybrid measurements were performed in a clinical catheter lab. Figure 6 shows initial results of simultaneous experiments with X-rays (Azurion 7 M 20 Flex, Philips, Germany) and iMPI under realistic pulsatile flow conditions (50 cm/s peak) and 4 frames per second real-time reconstruction (scanner parameters: currents *I*_CH1/2_ = 110 A, *I*_CH3_ = 75 A).

## Discussion

In this study a lightweight and portable MPI scanner dedicated for cardiovascular interventions of human-scale extremities is presented. This novel concept of field-free line generation allows real 3D movement of an FFL through the scanner without additional hardware requirements such as mechanical movement of scanner parts and/or sample bed^22–25^. Furthermore, the complexity of the scanner hardware is simplified by reducing the encoding method to projection imaging. This provides high flexibility and transportability because of reduced number of coils while offering good patient accessibility due to an open-design. In vitro real-time angiography, tracking of labeled endovascular instruments and experimental cardiovascular interventions such as a percutaneous transluminal angioplasty (PTA) in dynamic vascular phantoms were feasible. The presented data was acquired with 4 frames per second, which is commonly used in X-ray angiography. Since the acquisition time for each frame was 50 ms and the latency time until visualization was about 100 ms, up to 8 frame rates per second are possible. This is in the range of clinical fluoroscopy systems^40^. Simultaneous imaging with X-ray fluoroscopy, the gold standard for cardiovascular interventions, was feasible, as the iMPI scanner operates in unshielded environments and has a dedicated ‘X-ray window’ for potential hybrid imaging. The lightweight design of the iMPI scanner allows a direct assembling on the X-ray system providing multiplanar imaging from different directions. This improves the accessibility to the scanner, which is a paramount advantage of this design compared to conventional tomographic MPI scanners. For cardiovascular imaging, it may be necessary to display a second projection plane. For this purpose, the iMPI scanner could be mounted directly on a monoplane angiography system to be rotated around the patient analogous to conventional angiography. In principle, biplane angiography systems, such as those used for neurointerventions, would also be possible. Here, two pairs of tube detector units are installed perpendicular to each other. In this case, an additional X-ray window have to be introduced.

The absence of additional shielding components does greatly improve the accessibility to a patient in the scanner. However, external background noise from active hardware components causes distinct SNR reduction, especially in a clinical catheterization laboratory. As known from low-field MRI devices, which often are operating without shielding techniques^41^, there are several approaches available for active suppression of electromagnetic interference (EMI) using additional sensors picking up the environmental background noise, e.g., EDITER^42^. First implementations are also available for MPI for active suppression of EMI, e.g., the excitation frequencies^43^.

An important feature of MPI scanners is the gradient strength of the magnetic field, which directly correlates with the spatial resolution^23^. The proposed iMPI concept can provide a gradient strength of about 0.36 T/m at a power consumption of 14 kW (0.25 T/m at 7.5 kW—70% system power), which allows a spatial resolution of about 5 mm^25^. The spatial resolution of MPI is distinctly lower than the submillimeter resolution of X-ray fluoroscopy but comes with the advantage of a tracer-based background-free signal with high SNR.

The used coil design causes an intense sound generation, especially channel 3 running with 2480 Hz, when operating the system at high currents (scanner parameters: currents *I*_CH1/2_ = 165 A, *I*_CH3_ = 110 A). This results from the home-built coils that can vibrate under the huge force generation pushing apart the windings although the coils are encapsulated with epoxy resin. For performing the images in this work, the power of the scanner has been reduced to about 70%. To overcome this issue, future coils have to be assembled with less windings and encapsulated in stronger materials to avoid vibrations.

To increase the spatial resolution, the hardware and the applied particle system has to be improved. Tay et al. showed that using a novel type of superferromagnetic particle system resulted in a ten-times higher spatial resolution and 40-times higher SNR^44^. From the hardware side, the magnetic field gradient can be increased by running the field generator with higher currents or using coils with more windings. Both approaches have their limitations and require a sophisticated power management and cooling design. High power approaches using extremely short pulses for the generation of high magnetic field gradients^29^ may partially overcome this issue. However, in this case the FFL might travel only once along the symmetry axis of the scanner leading to a low spatial sampling density. This might require more sophisticated data acquisition and processing strategies to generate highly resolved raw-images by interleaved FFL trajectories^31,45^ over multiple pulse cycles.

The traveling wave concept of the iMPI scanner^36,37^ offers several flexible approaches to improve signal generation. The use of advanced sequences operating TWMPI scanner types, e.g., by adjusting the phases between CH1 and CH2, results in a higher gradient and zoom-like effects in the reconstructed images^30^. By additional modulation of the channel amplitudes, the coverage of the scanning area can be shaped to reduce SAR and PNS on the one side and to overcome the signal attenuation issue in MPI^46^. From the hardware side, parallel data acquisition by acquiring the signal generated by multiple FFLs simultaneously can further increase the usable FOV to overcome the intrinsic maximum size defined by the distance between adjacent traveling FFLs of the presented scanner^47^.

Choosing suitable parameters for operating an MPI scanner is an important step and can influence the performance of such scanners substantially^48^. The ratio between the frequencies *f*_1_ = *f*_2_ and *f*_3_, for example, influences the uniformity and resolution of the coverage of the FOV within a dedicated time window. The choice of frequencies influences the image quality distinctly. Increasing the frequencies for instance will typically increase the SNR due to higher induction. Above a certain frequency, depending on the used tracer material, the SNR decreases again, and the image resolution can markedly deteriorate due to the finite Néel and Brownian relaxation times of the MNPs^49^. There are novel approaches available to increase the spatial resolution by sophisticated data processing^50^. However, if real-time capability in interventional MPI systems is required, reduced data rates and simplified signal processing might be necessary^33^. Rapid data acquisition and signal processing require the accurate knowledge of all scanner parameters and used particle system for optimal results. This hampers the usage of the above-mentioned features, e.g., frequency and phase adjusting or coil adjusting, since the parameter changes need to be accurately tracked. By real-time monitoring of the FFL (or FFP) trajectory, e.g., using additional feedback coils, and AI-based reconstruction techniques^51^, the image quality can be enhanced while maintaining the flexibility of the MPI scanner.

Compared to studies using small animal scanners with a smaller bore, visualization of vascular pathologies such as aneurysms and stenoses was less accurate. It is technically impossible to achieve the highest values for spatial and temporal resolution, sensitivity, and spatial coverage simultaneously at once. In this scanner concept designed for vascular interventions, the focus was on real-time visualization in an open-design. In future studies, other criteria such as spatial resolution may by optimized, e.g., by applying above-mentioned concepts.

In Fig. 4d, a pseudo stenosis of a vessel segment aligned perpendicular to the main vessel direction is presented. Linear structures aligned into x-direction seem to vanish due to the fact, that by scanning with a field-free line along an elongated and homogeneous distribution no signal is generated. To overcome this issue, either the field-free line could be rotated by mechanical rotation of the gantry or scanner^23^.

The procedure of an experimental PTA was successfully performed, analogous to recently published studies using small animal scanners. In vivo applications would require more sophisticated interventional devices than the experimental ones in this study^52^. The signaling wire components would need to be coated with a biocompatible hydrophilic polymer coating to ensure low-friction maneuverability^17^.

As in MRI, for patient safety PNS and SAR limits must be considered^26^. By decreasing the magnetic field *B* and the frequency *f*, the SAR also decreases substantially. Since the magnetic field amplitude increases in human-sized systems due to the size of the FOV and the desired magnetic field gradient, the frequency has to be reduced. This also has a positive effect on PNS values, which are more pronounced in MPI scanners of this bore size. However, these limitations seem to hinder the upscaling of MPI scanners to human size with magnetic field gradients and frequencies known from preclinical studies. These problems may be overcome by additional techniques, such as scanning smaller areas with high gradients, which is similar to the focus field approach^53^.

In addition, the heating of ferromagnetic devices by alternating magnetic fields must be taken into account. Several studies regarding the heating behavior of endovascular devices, such as metal stents, showed that safe use is generally possible^54^.

The parameters for sensitivity and spatial resolution strongly depend on the application, e.g., for tracking labeled instruments within an MPI scanner, only a low spatial resolution and sensitivity is required. The instrument can be prepared with one spot consisting of highly concentrated material and the localization of one single point within the MPI scanner does not require a high spatial resolution^55^. But this can limit the examination of complex vasculatures like bifurcations or trifurcations. For the visualization of bolus-tracking, the sensitivity depending on the injected concentration of the tracer must be sufficiently high. But the spatial resolution needs necessarily to be high enough for the grading of vessel occlusions (stenoses). Even with a spatial resolution below the structural behavior, a quantification of vascular stenosis via signal attenuation along the vessel is possible^56^. Depending on the desired application, the iMPI design can cover different scenarios supporting X-ray machines.

The results of simultaneous iMPI and X-ray measurements are shown in Fig. 6 indicating a selected time point where the bolus travels through the vessel phantom. In the X-ray image, strong line artifacts are visible, which result from an interaction between the strong magnetic fields and the detector of the X-ray system.

The positioning of the point-like samples used for dilution series experiments (Fig. 2) has no influence on the results. All images were reconstructed using the same pre-calculated system matrix, but different numbers of harmonics have been collected from the Fourier transformed dataset for reconstruction. This specific peak-picking process is performed by thresholding peaks with signals less than SNR = 2. The graph in Fig. 2 shows the signal behavior at the first sideband frequency of the 5^th^ higher harmonic (5 × *f*_3_ + 2 × *f*_1_ = 12,520 Hz). The 3^rd^ higher harmonic band should show a higher amplitude but for imaging it is necessary to capture at least the next higher harmonic band, too.

The reconstruction method for the images used in this work is based on the image-based system matrix approach^46^, which allows to decouple the reconstruction process from the scanner hardware using a gridding process for the generation of raw-images as an intermediate step. This can be useful when working with instabilities in the hardware caused by coupling of the coils or for real-time reconstruction and visualization. A direct Fourier-based reconstruction method using spectral information for the system matrix can also be implemented^57^. Performing system matrix reconstruction, multiple artifacts on the final image can occur especially at the edges. This can be overcome by overscanning the FOV and adapting the system matric parameters^58^.

Limitations: the current proof-of-concept study has a number of limitations. The number of application examples was small, no quantitative analysis was performend and there was no systematic comparison with other imaging modalities such as X-ray based angiography, interventional MRI or intravascular ultrasound. The effects of the orientation of the vessel models along the grid and the effects of vessel size were not investigated. The exemplary vessel models tested were not quite to scale and slightly larger than the corresponding human vessels, which could affect signal intensity. Image quality in relation to motion was not explicitly investigated in this study but did not appear to have a significant impact during the experiments. Although the basic workflow of conventional angiography and interventions could be well replicated, there is still no approved intra-arterial MNP tracer dedicated for MPI available at the moment. However, therea are MNP-based MRI contrast agents for use in humans that can be visualized with MPI^58^. The limited gradient strength of this type of scanner leads to limitations in spatial resolution, which may affect diagnostic accuracy. Since the main problem here is not the hardware itself, but rather the PNS limitation, future scanner systems need to further reduce excitation frequencies and scan ranges. Combining multiple specific sequences should optimize the imaging process by selectively scanning the areas of interest^59^. Additional flexibility in the design of transmitting and receiving coils will overcome the current SNR challenges.

## Conclusion

The presented iMPI system is a human-scale scanner concept for real-time image-guided vascular interventions. The feasibility of such a system was demonstrated from initial scanner characterizations to experimental interventions in dynamic vascular models. The possibility of simultaneous hybrid use in combination with gold standard X-ray technology could accelerate translation to clinical use in vascular interventions to decrease ionizing radiation levels for patients and clinical staff and reduce the use of potentially nephrotoxic contrast agents. iMPI holds particular promise for endovascular interventions without ionizing radiation, which will enable broader use of these treatment tools without extensive protective measures in the field of (cardio-)vascular diseases.

## Methods

### Lightweight concept for human-sized MPI systems

The spatial encoding in Magnetic Particle Imaging (MPI) follows two principles: Field-free point (FFP)^6^ and field-free line (FFL)^35^. Both approaches have their specific advantages and disadvantages. The classic FFP design provides fast and sensitive imaging with a small FOV^14^. The basic FFL concept is characterized by very high sensitivity but imposes rather complex requirements on the field generation hardware, which limits its use for fast 3D imaging^23,60^. An alternative approach is the traveling wave MPI concept (TWMPI)^37,61^, which uses multiple coils for the dynamic generation of the required magnetic fields^36,37^. This flexible approach provides adjustable imaging features^30^ and fast imaging within a large FOV^32^ at the cost of lower signal-to-noise ratio (SNR).

### SAR and PNS

Safety limits of specific absorption rate (SAR), also known as tissue heating, and peripheral nerve stimulation (PNS), also known as magnetostimulation, determine the optimal scan parameters such as magnetic field strength and frequency or spatial resolution and scanning speed respectively.

The SAR limit regulates the energy, which may be applied in tissue (typically 4 W/kg)^62^. It depends on the magnetic field and the frequency (SAR scales with *f*^2^*B*^2^). For the given geometry and desired magnetic field gradient, magnetic field amplitudes for CH1/2 as well as CH3 of about 35 mT are required to steer the FFL through the FOV. The chosen frequencies for CH1/2 of *f*_1_ = *f*_2_ = 60 Hz and *f*_3_ = 2.480 Hz for CH3 cause a SAR of about 0.15 W/kg, which is far below the limit and allows to further increase the magnetic field gradient if necessary^26^.

For drive frequencies below 42 kHz, PNS is the dominant safety concern for MPI. PNS causes a sensory response in the muscle, described as a tingling, poking or twitching sensation. It is attributed to the electric field induced across neurons due to an applied oscillating magnetic field (magnetostimulation)^63^. With the fundamental law of magnetostimulation (FLM) as a function of frequency

1 $${B}_{th,pp}=\Delta {B}_{min,pp}\left(1+\frac{1}{2 {\tau }_{c}f}\right),$$

the maximum peak-to-peak amplitude of the magnetic field reaching the PNS limit can be calculated. Here, $\Delta {B}_{min,pp}$ is the asymptotic threshold for frequencies going to infinity. The chronaxie time ${\tau }_{c}$ measures the time required to depolarize the nerves^64^. With experimental values for leg (radius of about 6 cm) of Δ*B*_min,pp_ = 47.5 ± 7.0 mT and *τ*_c_ = 295 ± 56 µs^26^ and for the given frequency of *f*_3_ = 2480 Hz, a peak-to-peak amplitude threshold of about 80 mT can be calculated, which is slightly over the amplitude of 70 mT the iMPI scanner hardware is operated.

### Imaging

**Figure 7:**
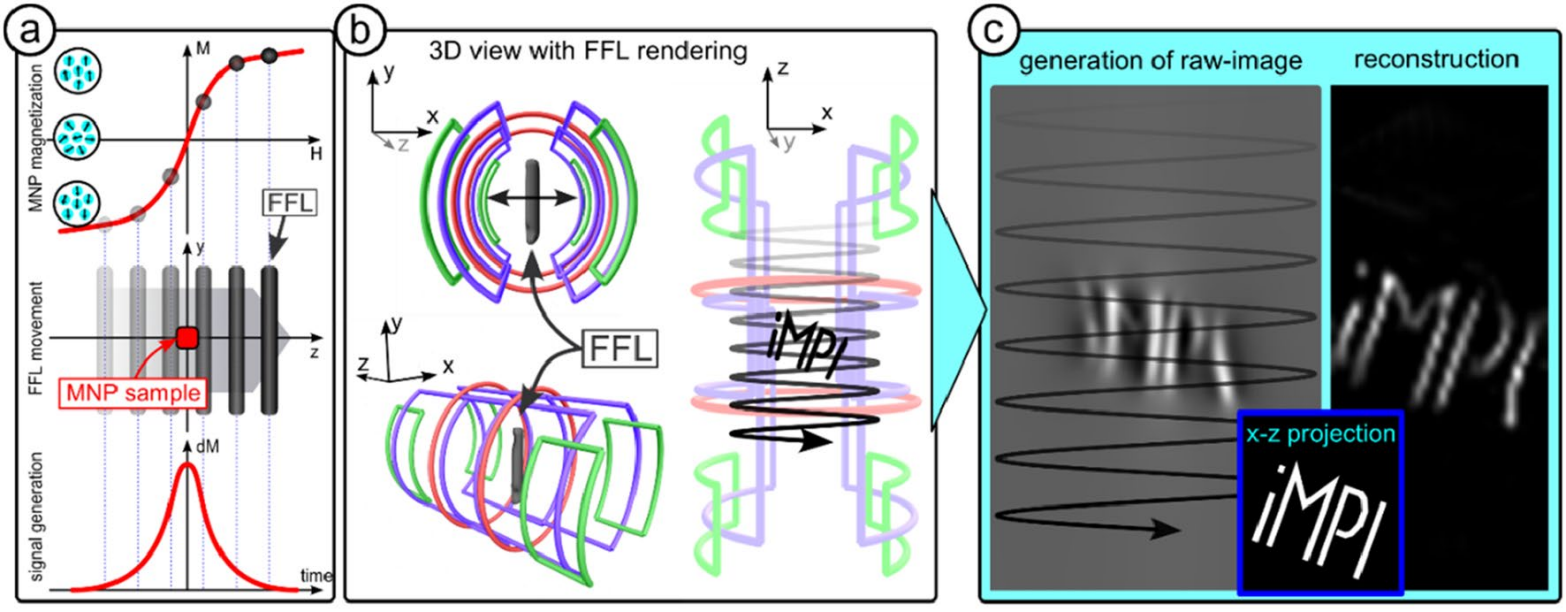
From signal generation to image reconstruction. (**a**) The magnetization of an MNP ensemble follows the nonlinear Langevin function. By steering rapidly, a strong magnetic field gradient, e.g., an FFL, over an MNP sample, the magnetization flips at the position of the FFL, and a signal *S* = d*M*/d*t* can be inductively measured. (**b**) The iMPI concept uses saddle-coils and solenoids for a dynamic generation and movement of an FFL (black surface rendering) along a sinusoidal trajectory within the x–z plane acquiring inductively the magnetization change of the MNP sample. (**d**) Since the position of the FFL is known, the 1D time signal can be co-registered and gridded point-by-point (line-wise following the *f*_3_ to *f*_1_ ratio) on a 2D image representing the x–z projection (raw-image) of the MNP distribution within the FOV. In a last step, the ‘iMPI’ letters serving as an example MNP distribution can be reconstructed in the final image.

Figure 7 describes the iMPI imaging&reconstruction process utilizing an FFL: Imaging in MPI is based on the nonlinear magnetization response of MNPs to time varying magnetic fields following the Langevin theory^6^. A strong magnetic field gradient in form of an FFL is generated and rapidly steered on a dedicated trajectory through the FOV. Since the magnetization of the MNP is saturated except in the vicinity of the zero-crossing areas of the magnetic field (FFL), the MNP magnetization flips when the field-free region of the gradient passes it in close vicinity. This rapid change in magnetization *M* is inductively measured using a receive coil resulting in the desired signal *S* = d*M*/d*t* (Fig. 7a).

The proposed iMPI concept uses the traveling wave approach for a dynamic generation and movement of an FFL along a sinusoidal trajectory covering the FOV in a projection image (x–z-plane). Figure 7b,c shows a simulation^65^ of a ‘iMPI’ lettering, which serves as an example for MNP distribution. The traveling FFL is oriented along the y-axis, the MPI signal is acquired from the entire line (FFL) resulting in a x–z projection. Since the trajectory of the FFL is well-known, the 1D signal can be co-registered and gridded point-by-point on a 2D image following the position of the FFL in space generating a raw-image. This raw-image shows the MNP distribution convolved by the scanner specific point-spread function (PSF)^57^. In a final step, the projection image can be reconstructed using image-based system matrix reconstruction^46^.

### From signal to the final image

**Figure 8:**
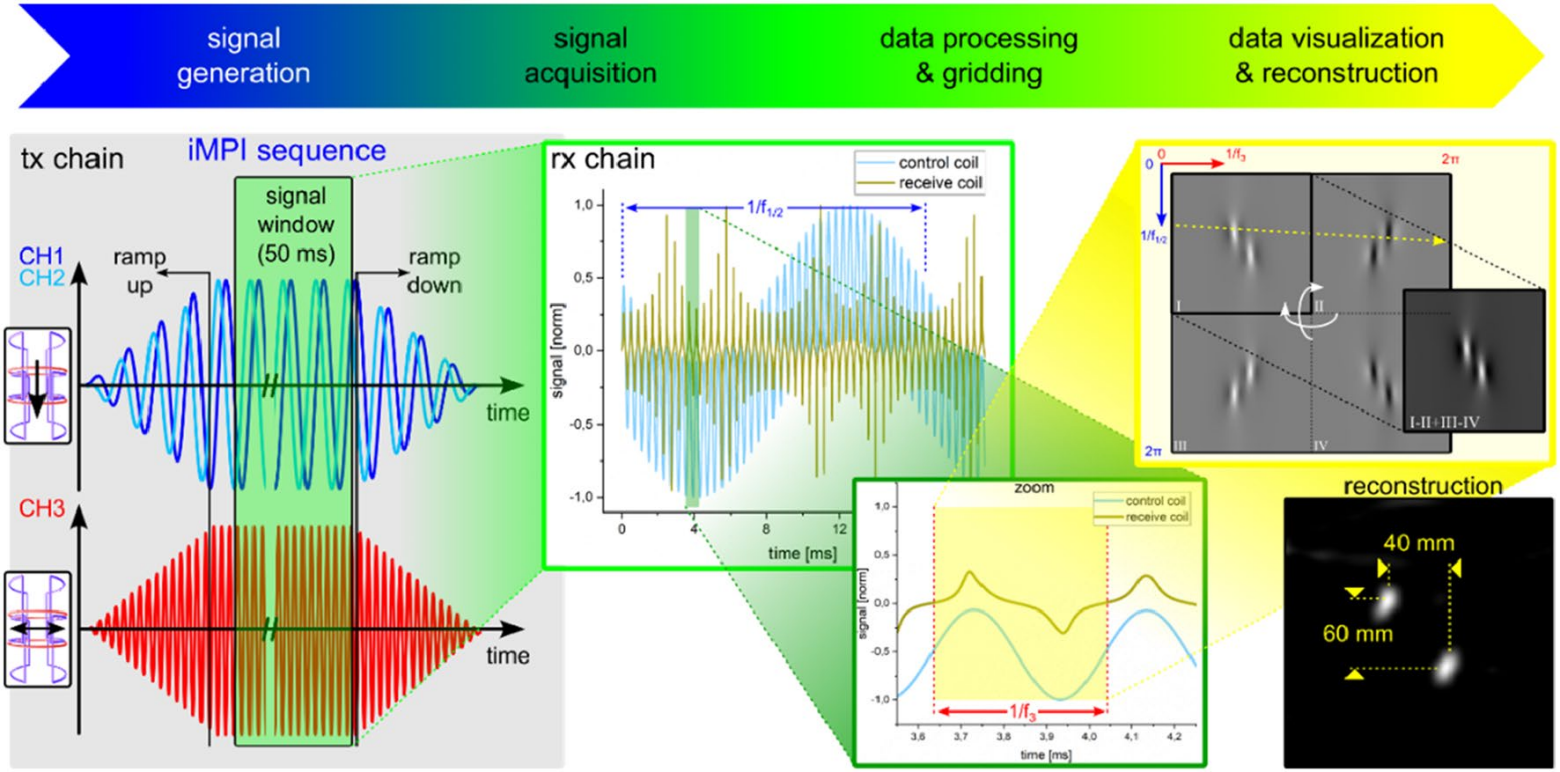
Sketch of the steps necessary for getting a fully reconstructed image: By starting the sequence, the selected waveforms for CH1/2 & CH3 are provided to the magnetic field generators. To ensure a transient-free magnetic field amplitude, the acquisition window starts with some delay after the finished ramp-ups. The digitized data set is processed and gridded on a 2D surface to create a raw-image before final reconstruction and visualization using a image-based system matrix approach^31,32,46^.

The acquired 1D data set consists of inductively measured changes of MNP magnetizations (*S* = d*M*/d*t*) and has to be processed (software filtering^31,33,37,47^) before raw-image preparation (see Fig. 8).

Since the position of the FFL within the scanner is known, the 1D data can be co-registered point-by-point on a 2D surface by filling up the 2D image line-by-line, where every line represents a full period of 1/*f*_3_. After a full period of 1/*f*_1/2_, the gridding process starts again at the beginning on a slightly different position, which depends on the ratio of *f*_3_ and *f*_1/2_. The result is a raw-image consisting of 4 times (2 times from left and right scanning and 2 times for each FFL sign) the MNP information from within the FOV convolved with a specific point-spread-function (PSF)^57^. Since all 4 areas show the same information, all areas can be folded together appropriately enhancing the SNR. With an appropriate reconstruction kernel, here an image-based system matrix, the final image can be calculated^54^. Since the entire raw-image generation uses processed (software-filtered, etc.) time data, noise in the raw-images can be reduced by selectively filtering harmonics from the spectrum with sufficient SNR (see section from signal to the final image).

### iMPI hardware components

The console (control unit) consists of hardware and software components, providing a graphical user interface (GUI) with automation- and reconstruction-software and additional hardware periphery, such as foot-pedal and input-pad, for fast and easy push-button scanner control. This allows full control on the transmit site from sequence selection to starting the experiment. In addition, the medical staff can use the iMPI scanner in a same way as known from X-ray systems. On the receive site, the acquisition window is triggered and all data processing steps from digitization over data gridding and reconstruction to real-time visualization are handled. For data reconstruction, an image-based system matrix reconstruction is used^46^, which provides robust image reconstruction results under real-time conditions^15,16,33^. The system matrices have been simulated in a prior step^65^ based on the iMPI calibration results and prepared for use in the reconstruction framework for real-time visualization.

#### Transmit chain

The transmit chain consists of three channels (CH) (Figs. 1, 10). Two are driving the main gradient (CH1 + CH2) creating a traveling FFL along the symmetry axis through the iMPI scanner and CH3 deflects the FFL to generate a projection image. Before setting up the transmit chain with the high-voltage filter components and amplifier cabinet, the frequencies for running CH1, CH2, and CH3 must be chosen appropriately. The adequate choice of frequencies here is *f*_1_ = *f*_2_ = 60 Hz and *f*_3_ = 2480 Hz, depended on multiple parameters, such as SAR and PNS limitation, desired temporal resolution and FOV coverage. With these frequency parameters, the components for each transmit channel (filters, coupling network, etc.) must be prepared. Finally, a channel decoupling was performed to guarantee a stable operation at high currents.

#### Power consumption and cooling

As stated before, the field generation for the desired gradient strength requires a high current to drive the gradient coils (CH1 & CH2) as well as the drive coil (CH3). With currents for CH1/2 of *I*_CH1/2_ = 165 A each and *I*_CH3_ = 110 A for CH3 and a measured electrical resistance of *R*_CH1/2_ = 0.5 Ω and *R*_CH3_ = 0.6 Ω, an effective power consumption can be estimated to *P*_eff,CH1/2_ = 1/2∙*R*_CH1/2_∙*I*^*2*^_CH1/2_≈6.6 kW and *P*_eff,CH3_ = 3.5 kW.

To get an idea about the temperature raise within each measurement, the following calculations can be used for estimation. Since the coils mostly consists of copper, in the first step, the mass of copper is calculated for each coil using *m*_Co_ = *V*_Co_∙*ρ*_Co_ = *A*_wire_∙*l*_wire_∙*ρ*_Co_, where *A*_wire_ = π∙400∙0.05^2^ mm^2^ = π mm^2^ is the cross section of the used litz wire (Rupalit 400 × 0.1, PACK LitzWire, Germany), *l*_wire,CH1/2_≈90 m and *I*_wire,CH3_≈105 m are the lengths for the coils and *ρ*_Co_ = 8.96 g/cm^3^ is the density of copper. Using the formula *P*∙Δ*t* = *m*_Co_∙*c*_Co_∙Δ*T*, where *c*_Co_ = 0.383 J/(g∙K) is the specific heat capacity of copper and Δ*t* = 0.06 s is the duty cycle for a single burst, and the calculated values, the temperature raise Δ*T* per image can be estimated to Δ*T*_CH1/2_≈0.41 K and Δ*T*_CH3_ = 0.18 K.

Since the iMPI scanner does not have an active cooling system, an estimation of the air-cooling capability based on convection is given in the following. Using the formula for the heat transfer *Q*/*t* = *α*∙*A*∙Δ*T* with the coil areas *A*_CH1/2_≈0.168 m^2^ and *A*_CH3_≈0.171 m^2^, an estimated temperature difference Δ*T* = 50 K between a heated coil and air temperature, and a heat transfer coefficient *α*_free_≈10 W/m^2^/K for convection driven cooling and *α*_forced_≈30 W/(m^2^∙K) for forced air flow using a ventilator, the temperature decrease per second can be estimated to Δ*T*_cooling,CH1/2_≈0.10…0.30 K and Δ*T*_cooling,CH3_≈0.09…0.27 K.

Thus, a continuous measurement with the iMPI scanner is possible with a framerate of about 0.5 to 1. Running at a framerate of 4, the iMPI scanner can be operated continuously up to 45 s before it would need to cool down.

#### Modular receive chain

**Figure 9:**
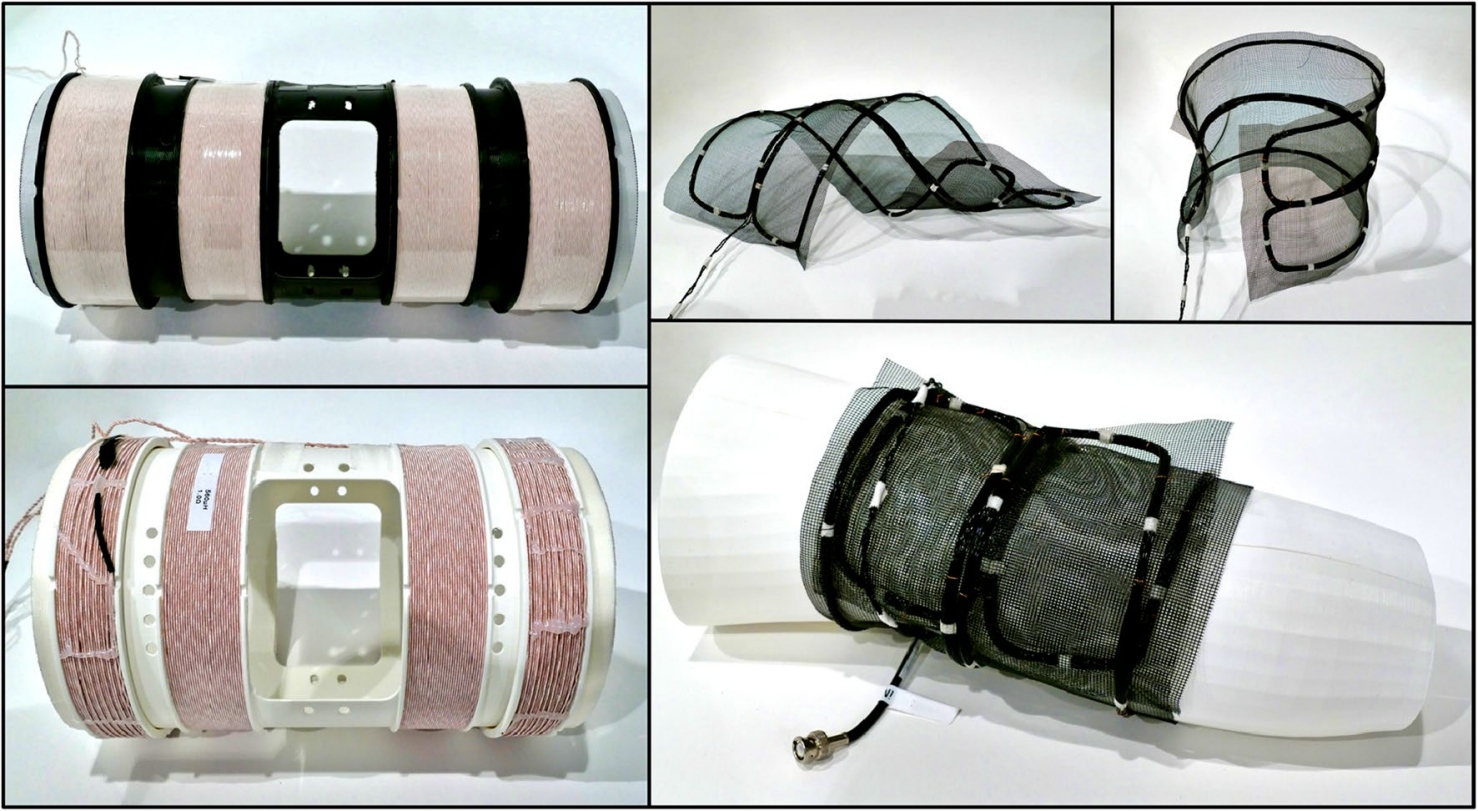
Two different approaches for receive coils are demonstrated: left: the rigid-body receive coils with different diameters (*d*_black_ = 11 cm, *d*_white_ = 18 cm) provide four coils each to be used as gradiometer. Both provide a dedicated X-ray window. Right: a flexible receive coil consisting of two racetrack coils assembled on a flexible material.

**Figure 10:**
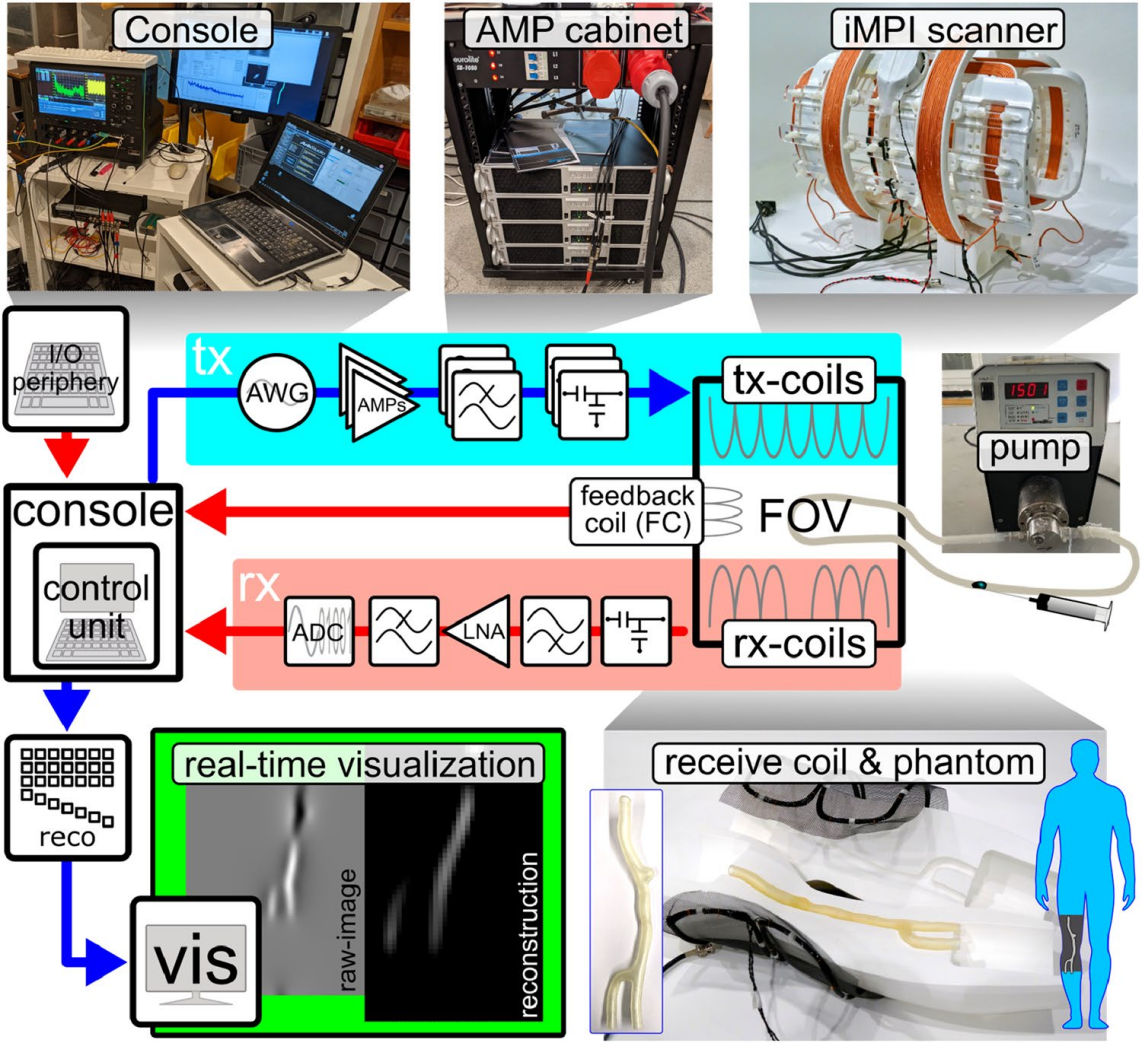
Overview of the experimental iMPI setup showing additional peripheral devices required for operating the iMPI scanner: The console consists of a control unit, which handles user inputs and coordinates the sequences for magnetic field generation, data acquisition, processing, and visualization. On the *transmit side*, an arbitrary wave generator (AWG) generates the designed sequence. An AMP-cabinet amplifies the sequence signals for high magnetic fields required for encoding and phantom visualization. On the *receive side*, the signal is inductively received using a receive coil (rx), filtered and amplified by passive low- and high-pass filters as well as low-noise amplifiers (LNA), and digitized using an analog–digital converter (ADC). Further signal processing steps (reco) are required to process the data before visualization (vis). An additional feedback coil (FC) is used to monitor the field parameters of the scanner.

The receive chain consists of multiple signal filters (high-, and low-pass filters), which were customized to the scanners’ frequencies, and a customized broadband low-noise pre-amplifier (LNA). The receive chain was implemented as a modular system allowing the use of different receive coils dedicated for specific applications. Using an additional impedance matching network, the resonance of the receive coil can be adjusted (see Fig. 10). In Fig. 9, two different types of receive coils are shown: on the left, two rigid-body versions with different diameters consisting of four coils each. The outer two coils can be adjusted to suppress the excitation signal (gradiometric receive coil). The black coil provides an inner diameter of 11 cm and a FOV length of about 12 cm. The white coil provides an inner diameter of 18 cm and a FOV length of about 14 cm. Both coils provide a dedicated X-ray window to be used in simultaneous X-ray-MPI measurements.

The flexible receive coil shown on the right site provides two racetrack coils (length 56 cm and width 7 cm) arranged on a flexible material forming a gradiometric design. This allows the coil to be wrapped around the sample for better SNR.

### Experimental setup

After assembly of the iMPI scanner, the entire setup must be implemented. Figure 10 gives an overview about the parts connected to the scanner. The console serves as control unit managing the users input via additional periphery such as foot-pedal or graphical user interface, generating the sequences for driving the field generators (transmit chain) and triggering the signal acquisition (receive chain) as well as the data processing (image reconstruction) and visualization.

### MPI visible instruments and applied tracer

Commercially available endovascular instruments did not show any relevant signal or artifact in the iMPI scanner and had to be modified with special markers for visibility. Published methods^15–17,20,52^ for marking endovascular instruments provided too little signal in the case of the iMPI scanner, thus a short piece of magnetic wire (paperclip) was attached to the instruments (0.035-inch Radfocus M hydrophilic guidewire (Terumo, Tokyo, Japan), Visi-Pro balloon (37 mm, Medtronic, USA)) for this study (Fig. 3). With this modification, the SNR was high enough for real-time visualization of guide-wire and balloon.

As intravascular tracer agent for visualizing vascular pathologies as well as dynamic angiography experiments with bolus injections, an experimental MPI tracer has been used (Perimag plain, 130 nm hydrodynamic diameter, iron concentration 8.5 mg/ml (LOT:00520102–03), Micromod Partikeltechnologie GmbH, Germany)^66^.

### Vascular phantoms and dynamic bolus injection

Human-sized vascular phantoms of the popliteal region were created from real 3D CT data sets. The retrospective use of these data sets was approved by the local ethics committee, with informed consent waived. The vessel structures were extracted in a first step using a home-built framework providing an easy-to-use 3D graphical user interface^67^. After post-processing, the extracted models were prepared for 3D printing (Form3, Formlabs, USA) with a flexible material (Elastic 50A resin, Formlabs, USA) to simulate vascular elasticity^68^. For percutaneous transluminal angioplasty experiments, an adjustable stenosis phantom has been developed to simulate a realistic endovascular treatment. The diameter of the vessel lumen is about 8–10 mm. For technical reasons (handling of the vessel clip), the parent vessel was 10 mm in diameter, larger than a human popliteal vessel (5–6 mm). Using different vessel clips, the grade of the stenosis could be adjusted from 70% down to 35% over a length of about 30 mm. The vessel clips can be removed in a treatment simulation by applying a balloon dilatation under realistic conditions. Realistic flow conditions are emulated with a gear pump, which provides continuous and pulsatile flow.

For realistic boli within the area of interest, 1 ml Perimag (iron concentration of 8.5 mg/ml) has been injected about 50 cm before the sample using a connected tube. After injecting the bolus, additional water has been used to rinsing off the tracer. Considering a dose limit of 200 mg iron for an 85 kg person (2.5 mg iron per kg)^38^, the amount and concentration seems to be useful for intra-arterial bolus injection and tracking.

### Ethical statement

Human-sized phantoms have been obtained from anonymized patients with informed consent (approved by ethic commission of University Hospital Würzburg, No. 20210310 04). All methods were performed in accordance with the relevant guidelines and regulations. The supplementary video shows the authors Dr. Stefan Herz and Dr. Patrick Vogel.

## Supplementary Information

Supplementary Information 1.

Supplementary Information 2.

Supplementary Information 3.

Supplementary Information 4.

Supplementary Information 5.

Supplementary Information 6.

Supplementary Information 7.

Supplementary Information 8.

Supplementary Information 9.

Supplementary Information 10.

Supplementary Information 11.

Supplementary Information 12.

Supplementary Information 13.

Supplementary Video 1.

## Data Availability

Processed measurement data set for generating the graphs are available on zenodo.org (https://doi.org/10.5281/zenodo.7146758). Raw data are available on request form the corresponding author [P.V.].
