# Supplementary figures and images for: iMPI: portable human-sized magnetic particle imaging scanner for real-time endovascular interventions
Source: Sci Rep. 2023 Jun 28;13:10472. doi: 10.1038/s41598-023-37351-2 (PMC10307843; doi:10.1038/s41598-023-37351-2)

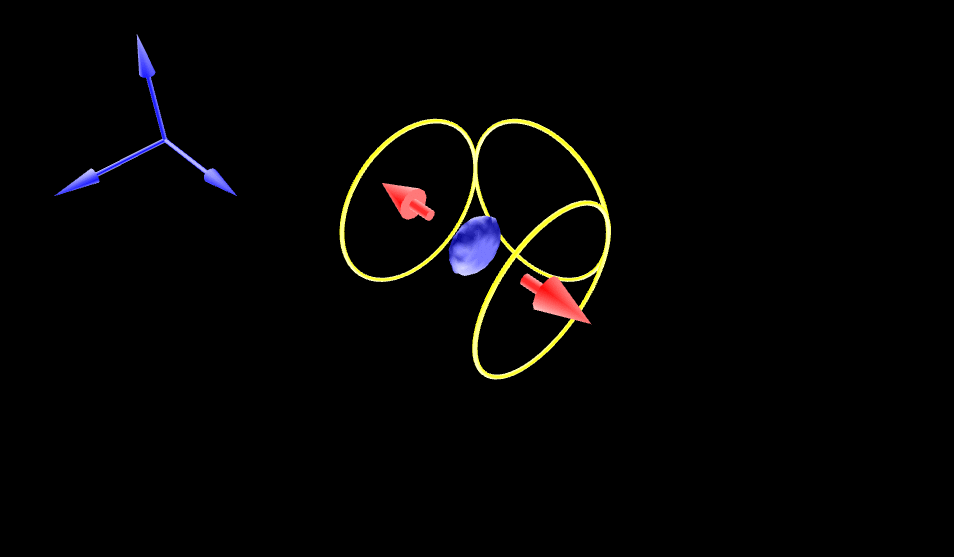

Supplement: Supplementary file 1 — Supplementary Information 1. [file 41598_2023_37351_MOESM1_ESM.gif]

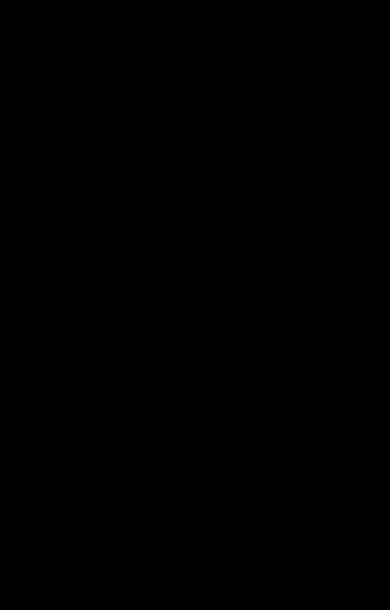

Supplement: Supplementary file 3 — Supplementary Information 3. [file 41598_2023_37351_MOESM3_ESM.gif]

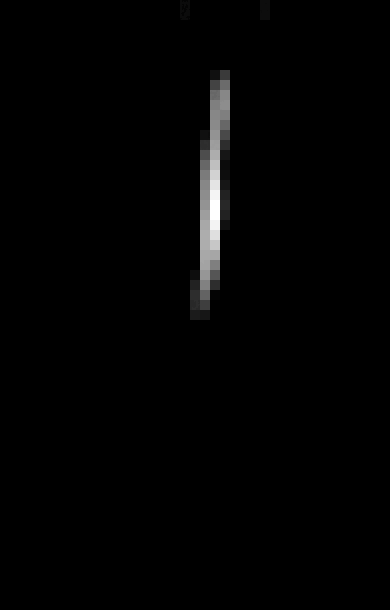

Supplement: Supplementary file 4 — Supplementary Information 4. [file 41598_2023_37351_MOESM4_ESM.gif]

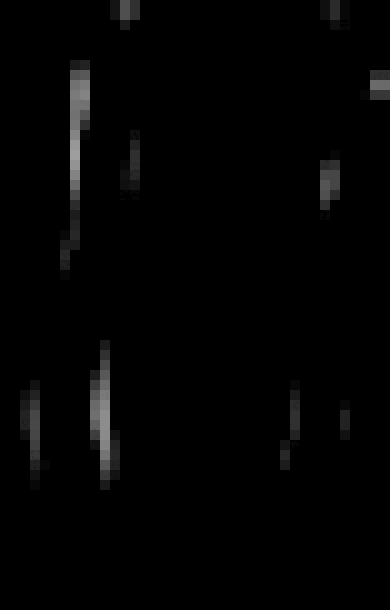

Supplement: Supplementary file 5 — Supplementary Information 5. [file 41598_2023_37351_MOESM5_ESM.gif]

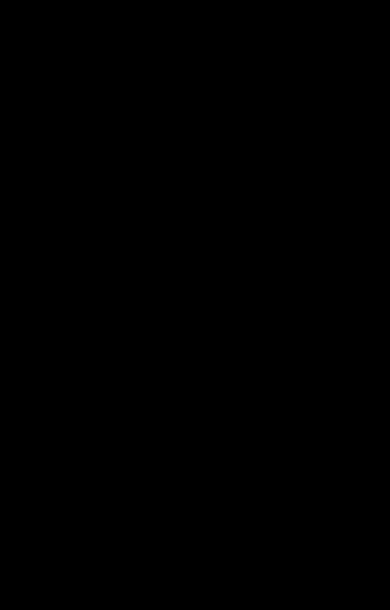

Supplement: Supplementary file 6 — Supplementary Information 6. [file 41598_2023_37351_MOESM6_ESM.gif]

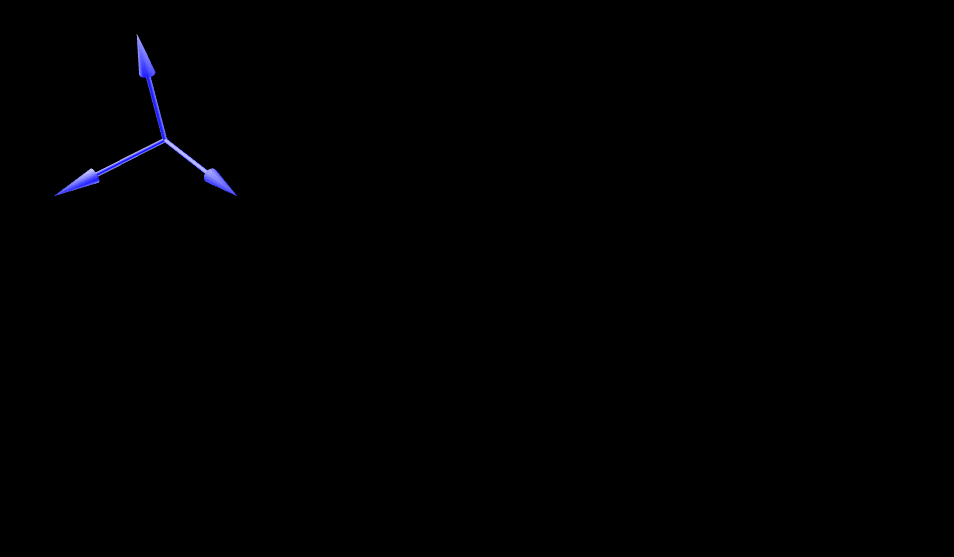

Supplement: Supplementary file 7 — Supplementary Information 7. [file 41598_2023_37351_MOESM7_ESM.gif]

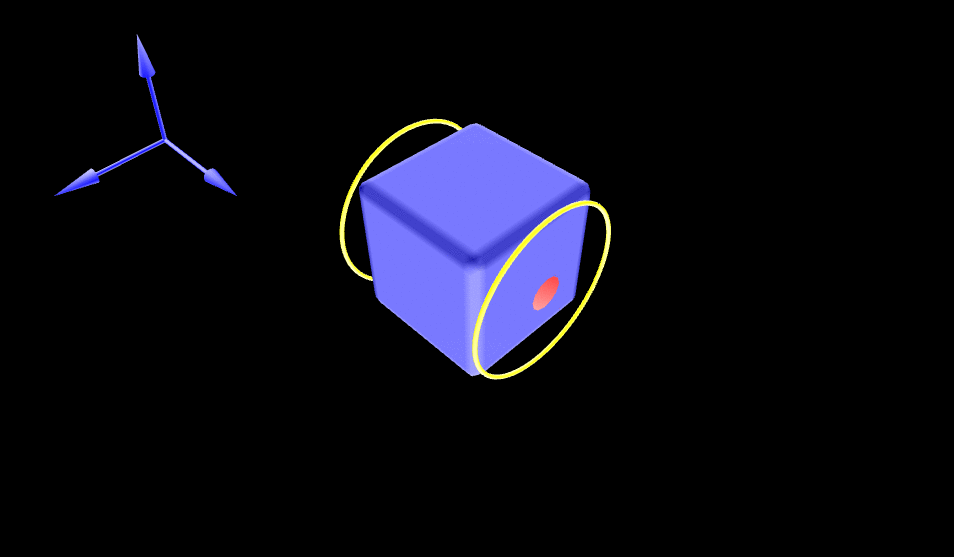

Supplement: Supplementary file 8 — Supplementary Information 8. [file 41598_2023_37351_MOESM8_ESM.gif]

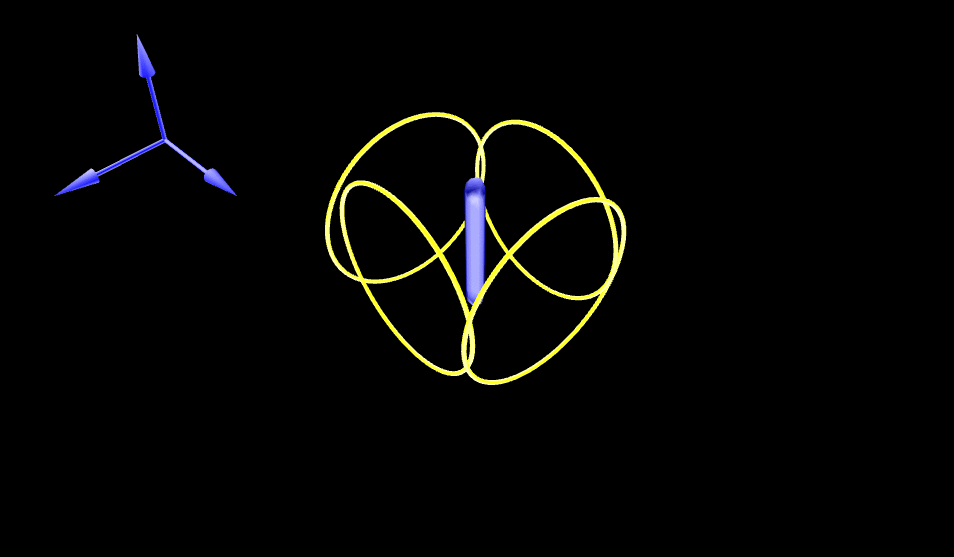

Supplement: Supplementary file 9 — Supplementary Information 9. [file 41598_2023_37351_MOESM9_ESM.gif]

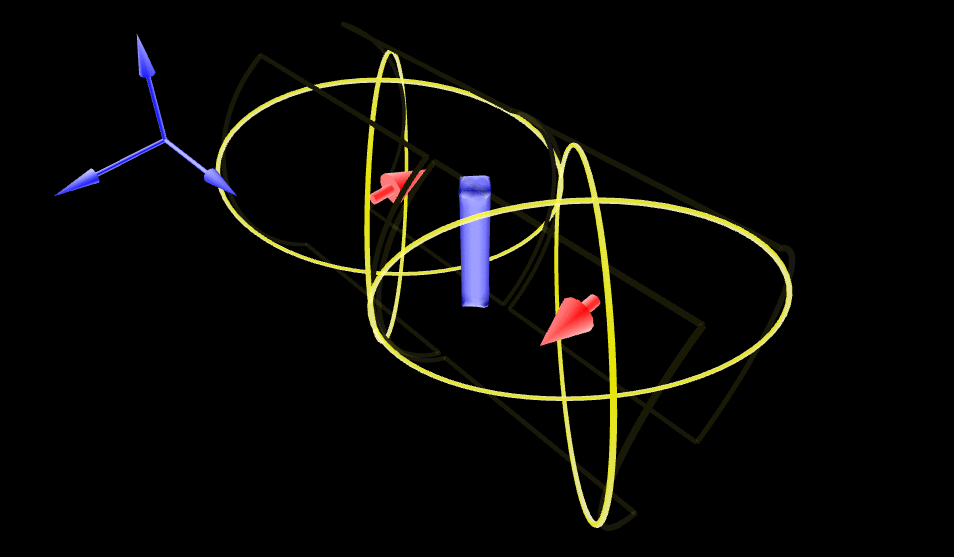

Supplement: Supplementary file 10 — Supplementary Information 10. [file 41598_2023_37351_MOESM10_ESM.gif]

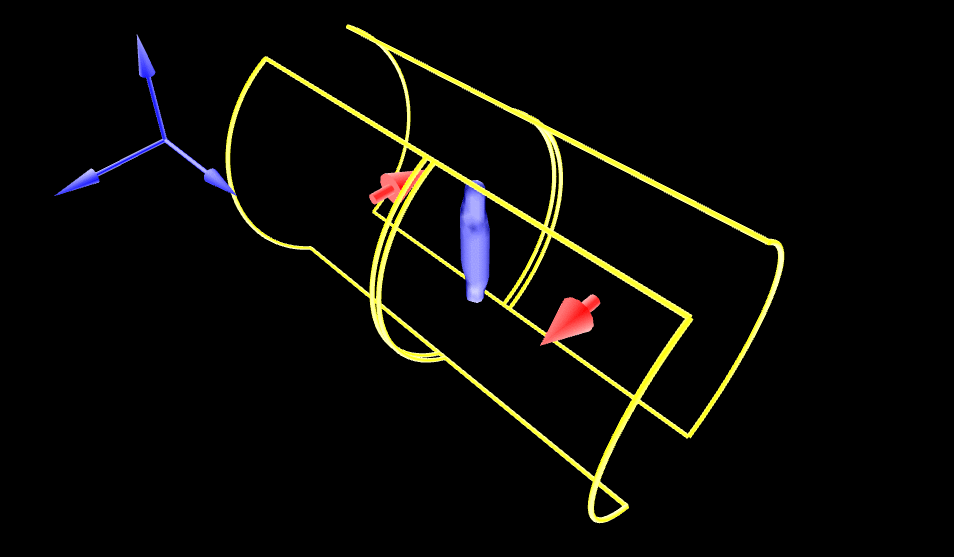

Supplement: Supplementary file 11 — Supplementary Information 11. [file 41598_2023_37351_MOESM11_ESM.gif]

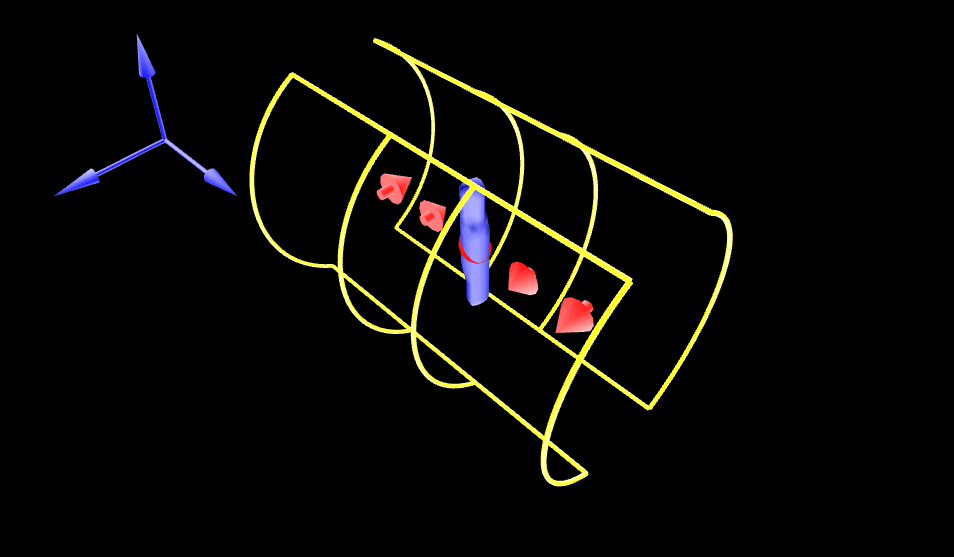

Supplement: Supplementary file 12 — Supplementary Information 12. [file 41598_2023_37351_MOESM12_ESM.gif]

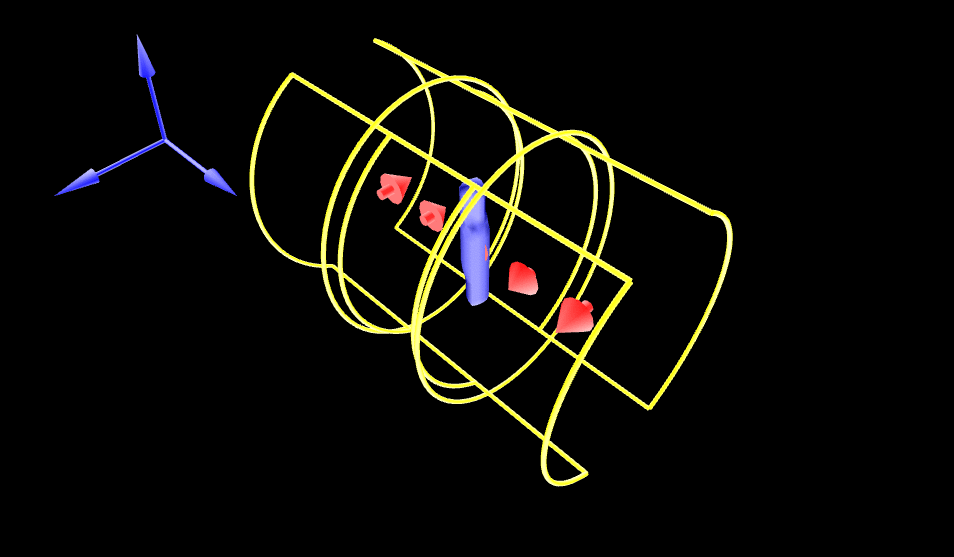

Supplement: Supplementary file 13 — Supplementary Information 13. [file 41598_2023_37351_MOESM13_ESM.gif]
